# Effect of temperature on the accumulation of marine biogenic gels in the surface microlayer near the outlet of nuclear power plants and adjacent areas in the Daya Bay, China

**DOI:** 10.1371/journal.pone.0198735

**Published:** 2018-06-11

**Authors:** Wei-zhong Yue, Cui-ci Sun, Ping Shi, Anja Engel, You-shao Wang, Wei-Hong He

**Affiliations:** 1 State Key Laboratory of Tropical Oceanography, South China Sea Institute of Oceanology, Chinese Academy of Sciences, Guangzhou, China; 2 Daya Bay Marine Biology Research Station, Chinese Academy of Sciences, Shenzhen, China; 3 GEOMAR Helmholtz Centre for Ocean Research Kiel, Kiel, Germany; 4 South China Sea Institute of Oceanology, Chinese Academy of Sciences, Guangzhou, China; University of California, Merced, UNITED STATES

## Abstract

The surface microlayer (SML) in marine systems is often characterized by an enrichment of biogenic, gel-like particles, such as the polysaccharide-containing transparent exopolymer particles (TEP) and the protein-containing Coomassie stainable particles (CSP). This study investigated the distribution of TEP and CSP, in the SML and underlying water, as well as their bio-physical controlling factors in Daya Bay, an area impacted by warm discharge from two Nuclear power plants (Npp’s) and aquaculture during a research cruise in July 2014. The SML had higher proportions of cyanobacteria and of pico-size Chl *a* contrast to the underlayer water, particularly at the nearest outlet station characterized by higher temperature. Diatoms, dinoflagellates and chlorophyll *a* were depleted in the SML. Both CSP and TEP abundance and total area were enriched in the SML relative to the underlying water, with enrichment factors (EFs) of 1.5–3.4 for CSP numbers and 1.32–3.2 for TEP numbers. Although TEP and CSP showed highest concentration in the region where high productivity and high nutrient concertation were observed, EFs of gels and of dissolved organic carbon (DOC) and dissolved acidic polysaccharide (> 1 kDa), exhibited higher values near the outlet of the Npp’s than in the adjacent waters. The positive relation between EF’s of gels and temperature and the enrichment of cyanobacteria in the SML may be indicative of future conditions in a warmer ocean, suggesting potential effects on adjusting phytoplankton community, biogenic element cycling and air-sea exchange processes.

## Introduction

The sea surface microlayer (SML) is the topmost 1–1000μm of the ocean surface. In recent years, enrichment of biogenic marine gel particles in the SML have gained increasing attention as these gelatinous compounds may play an important role in microbial processes and carbon cycling in the ocean as well as potentially affecting of the exchange of gas, heat and mass between the ocean and atmosphere [1–4]. Marine gels, such as transparent exopolymer particles (TEP, polysaccharidic) and Coomassie stainable particles (CSP, proteinaceous) [5, 6], are formed by exopolymers of polysaccharidic or proteinaceous composition mostly derived from marine microorganisms during exudation, degradation and lytic processes [7–9]. Trapped by ascending bubbles, the sticky gels can be carried upward through the water column to the SML, or form from dissolved precursors directly at the air-sea interface during surface wave action [10]. It has been suggested that the presence of abundant biogenic gels in the SML can influence gas exchange between air and sea [4] and provide an important source for marine primary organic aerosols [11, 12]. Additionally, due to the sticky properties of gels, they can aggregate both live and dead plankton, as well as mineral particles and therefore represent hotspots of microbial activity in the SML [13, 14].

The major factors determining the production of biogenic gels and their precursors in the water column include the physiological state of the phytoplankton cell and phytoplankton community composition as well as environmental growth conditions [15–17]. Therefore, feedbacks of phytoplankton in the surface ocean to environmental changes could be reflected in the accumulation of marine gels in the SML [18]. Lass, Bange [19] showed that seasonal variations of abundant carbohydrate-rich polymeric material, such as TEP precursors, in the SML were related to a combination of phytoplankton abundance and photochemical and/or microbial reworking of organic matter. High TEP production has often been observed towards the end of algal blooms or in cultures of nutrient-stressed phytoplankton [20, 21]. For CSP abundance, significant increase by initial NO_3_ supply was observed during mesocosm experiments [22]. In contrast to gel production in the water column, enrichments of dissolved protein fractions in the SML have been inversely related to trophic status, i.e. higher enrichments were observed in oligotrophic open ocean areas compared to more productive coastal waters [23]. CSP concentration might be influenced in the SML by the same processes as in bulk seawater (production by or leaching from phytoplankton and bacteria; microbial degradation; photodegradation), but at different rates[24–26]. The different rates between SML and bulk water might yield a different CSP turnover time and a complex pattern of enrichment in the microlayer[23]. While these results showed that the production of biogenic gels by phytoplankton was tightly related to the tropic status, it is yet difficult to predict the impact of nutrients supply on the enrichments of the gels in the SML due to co-effects on phytoplankton community structure, in particular as we still lack information on the controlling factors for CSP formation and accumulation in the SML and as well as in the water column.

Besides nutrients supply, temperature has been suggested to affect the production of TEP by phytoplankton [27, 28]. Thereby, the effect of elevated temperature on the production of gels depended on the geographical temperature variability and on the interactive effects between different environmental conditions, i.e. under moderate or cool temperature condition, the production of TEP was promoted by elevated temperature through an increasing amount of exudates released by phytoplankton when nutrients become limiting [29–32]. But, rising temperature could also result in an earlier onset of TEP degradation by heterotrophic bacterial communities, which could counter act the enhanced TEP production[33]. In the low latitude ocean characterized by higher temperature, elevated temperature is expected to result in increased stratification of the upper water column diminishing upward nutrient supply [34, 35]. In the context of global warming, it is increasingly important to understand the dependence of temperature on the accumulation of marine gels in the SML, due to the potential importance of the biogenic gels in modifying exchange processes across the air and sea interface as well as their well-known roles in microbial processes and carbon cycling.

Daya Bay (DYB) is one of the largest bays along the coast of southern China. In the past decades, the rapid economic development and human activities in this area had a profound influence on the environment of the bay. The marine aquaculture industry has also been one of the important industries in this area since the 1980s. Dapeng Ao, Aotou, and the north part are mainly marine aquacultural areas. Dissolved inorganic nitrogen (DIN) increased from 1.53 to 5.40 μmol L^-1^ in DYB due to anthropogenic inputs in the period from 1985 to 2004. In contrast, dissolved inorganic phosphorous (P) decreased from 1.12 to 0.110 μmol L^-1^, probably as a result of ban-used detergency powder (contains phosphorous) in recent years, resulting the average ratio of N/P increased from 1.377 in 1985 to 49.09 in 2004 [36]. Two nuclear power plants (Npp), Daya Bay Npp (DNpp) and Ling'ao Npp (LNpp), are located on the western coast of DYB and started up in 1994 and 2002, respectively. Cooling sea water collected from the bottom was heated by LNpp and DNpp and taken through a common canal to discharge into DYB. For LNpp, total rated capacity of the facility is 4000 MW and uses about 220 m^3^ cooling water per second, and cooling water increased up to 9°C by heat exchange when passing the condensers. For DNpp, the total rated capacity is 1800 MW, and the condenser circulating system needs an intake and discharge flow of 95 m^3^s^-1^, with a temperature elevation of 10°C. The formation of a seasonal thermo-cline with temperature gradient of 0.3~1.72°Cm^-1^ in DYB occurs from May to October, and is especially pronounced between July and September [37]. The thermal discharge has a visible influence on the thermal stratification [38]. The difference in temperature between surface and bottom reached 5.98°C near the output of warm discharge, higher than a mean difference of 3.5°C in DYB [37]. This is expected to shallow the mixed layer depth from 6-8m at the mouth of DYB to 2-4m at the outlet of Npp. Furthermore, vertical mixing with deeper, nutrient-rich water was strongly diminished when no strong precipitation and runoff inputs were observed in summer, after the thermocline is established, which signals the onset of stratification [37]. Apart from the thermal discharge from the Npp, anthropogenic nutrients inputs impose stresses on water bodies in DYB, altering the phytoplankton community composition, biomass as well as the size of plankton, especially near the aquaculture farm areas [36, 39–41]. In addition, it has been shown that autotrophic phytoneuston, i.e. organisms inhabiting the SML, in DYB is a unique community jointly dominated by cyanobacteria and diatoms, and has a different community structure compared to phytoplankton in the underlying bulk water [42]. Changes in the autotrophic phytoneuston community may serve as indicators of the environmental changes in DYB, such as increasing water temperature, global warming, and nutrient supply. Thus, it is expected that these environmental changes influence the production and accumulation of gels in the SML as well as in the underlying water in DYB.

The aim of this study was to investigate how properties of the SML near the outlet of nuclear power plants in DYB, and specifically the impact of warm water discharge from Npp affect the phytoneuston community and distribution of biogenic gels in the SML. In view of the long-term (20-year) operation of the Npp and the accompanying increase of water temperature at the Npp site, the advantage of this study compared to other 'warming experiments' like bottle or mesocosms experiments is that we examined a natural community, which had time to adapt to a changed environmental situation.

## Materials and methods

### Ethics statement

No animals were collected for these surveys, and no sites required permits for general research during this research. This study did not involve endangered or protected species.

### Sites and sampling

DYB is a semi-enclosed bay in the northwestern part of the South China Sea. It lies to the east of the Pearl River Estuary, with an average depth of 11 m. The enclosed character of the bay reduces the rate of water exchange with open ocean water, reinforcing the exposure to elevated nutrient input. No large river discharges into the bay, however, there are more than ten seasonal streams flowing into the bay from a short distance along the coast. Fish and shell aquaculture are well developed, with cage culture industry widespread in the inner of Dapeng Bay. The East Guangdong upwelling transports cold water to DYB, and the thermal discharge from nuclear power plant increases the thermo-cline strength during the summer from July to August [38, 43].

**Fig 1:**
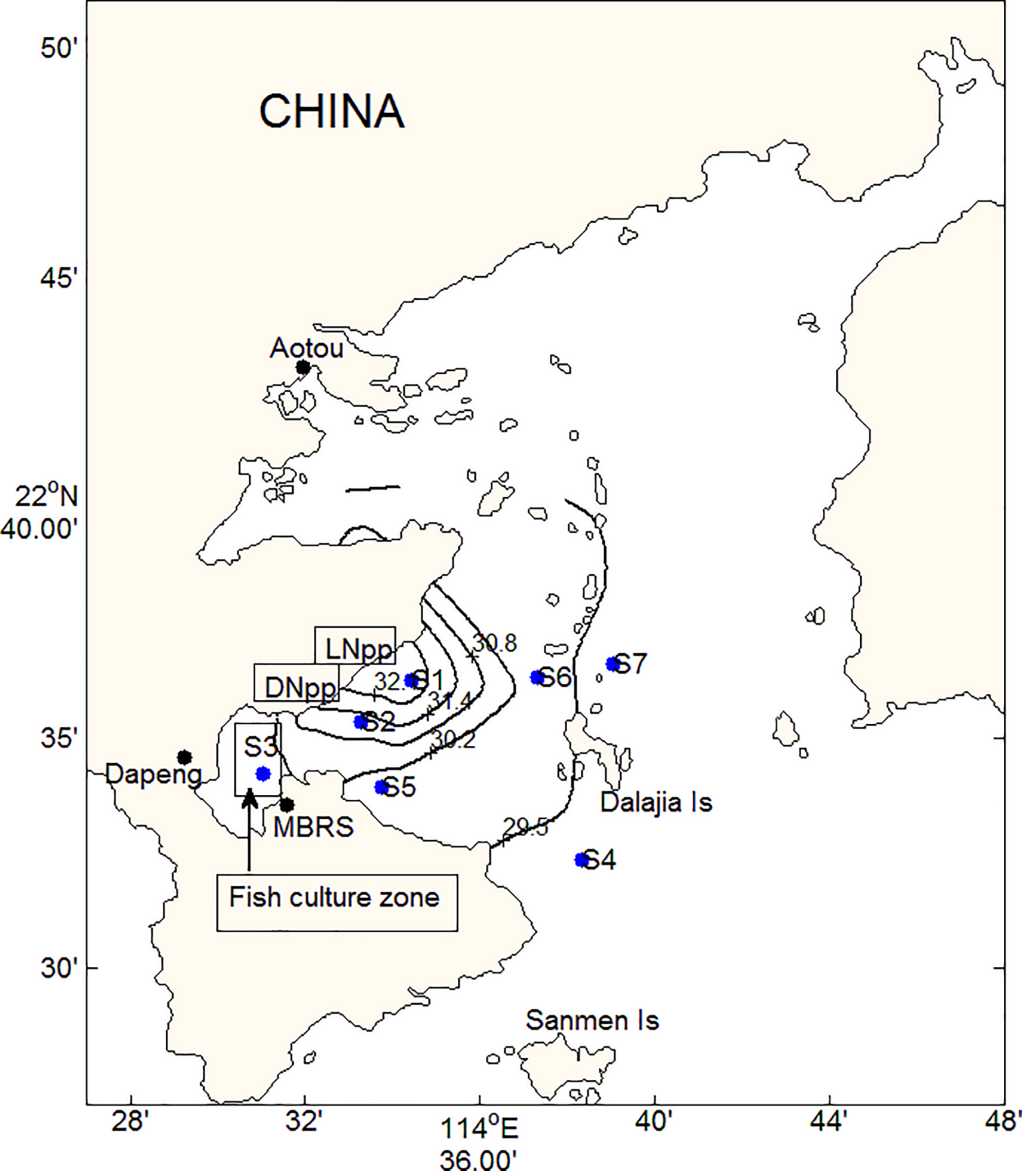
Sampling stations and surface temperature distribution in Daya Bay (DNpp, Daya Bay Nuclear Power Plant; LNpp, Lingao Nuclear Power Plant, MBRS, Daya Bay Marine Biology Research Station).

The cruise took place near the outlet of the thermal discharge and in the adjacent DYB area in July 2014 (Fig 1). At this time, surface seawater temperature was the highest during the year (range: 14.4–32.4°C with average of 22.4°C). Samples were collected from two sites (S1 and S2) close to the Npp and at various distances from the hot-water site (S3-S7) to compare properties of the SML with respect to seawater temperature. All sampling sites were located between 1.1 (S1) km and 12.2 km (S4) from the Npp. Long term monitoring data collected by the Marine Biological Research Station (MBRS) showed that surface temperature at S1 has increased by 2–3°C compared to S4 since the beginning of the operation of the Npp [38]. S3 was in the Dapeng Bay, where the water is generally less affected by oceanic water inflow, because of sluggish water circulation. At each sampling site, salinity, temperature, and pH were measured over the full water-column using a YSI 6600 sonde (YSI Environmental, USA).

Wind speed at 10 m above ground was retrieved from the DYB station database (http://dyb.cern.ac.cn/) for the time of sampling, acquired using an automated Vaisala Milos 520 weather station. The photosynthetically available radiation (PAR) and ultraviolet (UV) radiation were determined by Vaisala Milos 520. SML samples were collected using a glass plate sampler according to the original approach described by Harvey and Burzell [44] from a small boat. For each sample, the glass plate was vertically inserted into the water and withdrawn at a controlled rate of ~20 cm s^-1^. The sample, retained on the glass because of surface tension, was removed by a Teflon wiper. Samples were collected into acid cleaned (HCl, 10%) and Milli-Q washed glass bottles. Prior to sampling, both glass plate and wiper were washed with HCl (10%) and intensively rinsed with Milli-Q water. Between samplings, both instruments were copiously rinsed with seawater to minimize their contamination with alien material while handling or transporting the devices. Samples were collected as far upwind of the boat as possible and away from the path taken by the small boat to avoid contamination.

The apparent thickness (*d*) of the layer sampled with the glass plate can be determined as follows:

d=V/(A×n) (1)

where *V* is the SML volume collected, *A* is the sampling area of the glass plate and *n* is the number of dips. We will use *d* (μm) as an operational estimate for the thickness of the SML. At the same stations, after sampling the SML, samples from subsurface layer (SSL) were collected with a Niskin water sampler at 0.5 m below the surface, respectively. The SML thickness determined from a total of n = 7 samples, ranged from 41–58μm, with a mean value of 44.85±5.93 μm. This value is consistent with the previous observations with a glass plate at the same withdraw rate [45].

Results from the SML samples were compared to those of underlying water and expressed as enrichment factors (EF), defined as:

$$EF={\left(C\right)}_{SML}/{\left(C\right)}_{SSW}$$ (2)

Where (C) is the concentration of a given parameter in the SML or SSW water, respectively [46]. Enrichment of a component is generally indicated by EF> 1, depletion by EF< 1.

### Biological and chemical measurements

#### Inorganic nutrients and phytoplankton community structure

A 250-ml aliquot water was gently filtered (<100 mbar) through 20 μm nylon membrane (Millipore), 2.0 μm and 0.2 μm polycarbonate membrane filters (Millipore) using a serial filtration unit and fractionated into pico- (<2.0 μm), nano- (2.0–20.0 μm) and micro-phytoplankton (>20 μm) size fractions [47]. Chl *a* concentration was determined using fluorescence method after extraction in 90% acetone for 24 h. The filtrates were used for dissolved nutrient analysis, including DIN, (the total of nitrate, nitrite and ammonia) and DIP. Nutrient analysis was done in triplicate following Strickland and Parsons [48].

For phytoplankton counting, samples were fixed with pre-filtered paraformaldehyde (0.5% final concentration) for 30 min and flash frozen in liquid nitrogen, and stored at -80°C until analysis. Three major groups of phytoplankton, cyanobacterial (mainly *Synechococcus* spp.populations), and eukaryotic phytoplankton of pico-size were discriminated and enumerated based on their auto-fluorescence properties using a flow cytometer (Becton & Dickinson FACSCalibur) equipped with a laser emitting at 488 nm. *Synechococcus* spp. populations were distinguished principally by their orange fluorescence and separated from the distributions of other populations in the plot of red vs blue florescence. Picoeukaryotes always showed the largest red fluorescence and slide scatter. Cell counts were analyzed using BD CellQuest Pro-Software.

Samples for characterizing the microphytoplankton community composition were stored using 10% formalin (final conc. 1%). The concentrated sample was examined by inverted light microscopy at magnifications of 400x, after settling 25 mL in Utermöhl chambers for 24 h. Diatoms, dinoflagellates and cyanobacteria were enumerated in fields of view with 0.5 mm diameter.

#### Total organic carbon (TOC), dissolved organic carbon (DOC) and particulate organic carbon (POC)

Samples for TOC and DOC (20 mL) were collected in combusted glass ampoules, DOC after filtration through combusted GF/F filters (8 h, 500°C). Samples were acidified with 80 μL of 85% phosphoric acid, heat sealed immediately, and stored at 4°C in the dark until analysis. DOC and TOC samples were analyzed by applying the high-temperature catalytic oxidation method (TOC-VCSH, Shimadzu) modified from Sugimura and Suzuki (1988). Potassium phthalate standard calibration was conducted over the range 0 to 250 μmol C L^-1^. The blank of the analytical system was between 5 and 10 μmol C L^-1^ and the standard deviation was less than 3% of the mean of triplicate measurements. Particulate organic carbon (POC) was determined as the difference between TOC and DOC.

#### Gel particles

Total area, particle numbers and equivalent spherical diameter (*dp*) of gel particles were determined by microscopy [49]. 2 to 10 mL were gently filtered (<150mbar) onto 25mm Nuclepore membrane filters (0.4 μm pore size, Whatman Ltd.), stained with 1 mL Alcian Blue solution for TEP and 1mL Coomassie Brilliant Blue G (CBBG) working solution for CSP. Excessive dye was removed by rinsing the filter with Milli-Q water. Blank filters for gel particles were taken using Milli-Q water. Filters were transferred onto Cytoclear slides and stored at -20°C until microscopically analysis. For each filter, thirty images were randomly taken at ×200 magnification with a light microscope. An image-analysis software (Image J, US National Institutes of Health) was used to analyze particle numbers and area.

The size-frequency distribution of TEP and CSP gels was described by:

$$dN/d(dp)=kd_{p}^{\delta }$$ (3)

where d*N* is the number of particles per unit water volume in the size range *d*_*p*_ to(*dp* +d(*d*_*p*_)) [50]. The factor *k* is a constant that depends on the total number of particles per volume, and δ (δ< 0) describes the spectral slope of the size distribution. The less negative is δ, the greater is the fraction of larger gels. Both δ and *k* were derived from regressions of log[d*N*/d(*d*_*p*_)] versus log[*d*_*p*_].

For fractal scaling of particle size distribution, three linear regions with different slopes can be determined in accordance with the three collision mechanisms, Brownian motion, fluid shear and differential sedimentation [51]. Brownian motion controls small particles ranging from 0.1 to 2 μm. For the size of 2–60 μm, fluid shear is considered to dominate, and differential sedimentation becomes dominant for particles larger than 60 μm [52]. If larger gels-particle aggregates are denser than seawater due to higher proportion of attached solid particles in the coastal area, these larger particles would be expected to settle out of the SML, consequently reducing their abundance in the SML [16]. Thus, in this study, slope for all size gel particles was determined only for the size of 2–60μm ESD.

Concentrations of TEP_color_ (μg Gum Xanthan equivalents (Xeq.) L^-1^) were measured using the method of Passow and Alldredge[53]. Triplicate 40 ml samples were vacuum filtered (<200 mbar) onto 25 mm diameter, 0.4 μm polycarbonate filters (Millipore). Filters were stained for <5 s with 0.5 mL of 0.02% Alcian Blue 8GX (Amresco) in 0.06% acetic acid (pH 2.5) and then rinsed with 2.0 mL of deionized water. Alcian Blue-stained material was extracted from the filters with 6 mL of 80% sulfuric acid for 2 h on an oscillator. Absorbance of the supernatant fluid was measured spectrophotometrically at 787 nm. Alcian Blue absorption was calibrated using a Xanthan Gum solution (SIGMA) that was processed by tissue grinder and measured by weight.

Dissolved acidic polysaccharides (DAPS) were measured using an Alcian Blue staining method [54]. 20 ml samples were filtered through 0.2 μm pore-size syringe filter containing a surfactant-free cellulose acetate (SFCA) membrane. The filtrate was collected into sterile polyethylene centrifuge tubes, preserved with 0.2 mL formalin and stored refrigerated (4°C) until analysis. Alcian Blue can precipitate with salts. In order to remove inorganic salts interferences ([53, 54]), duplicate samples after shaking were desalinated by dialysis tubing with a molecular weight cut-off of 1000 Da (Spectra/Por 7 regenerated cellulose, Spectrum Laboratories) for approximately 20 h. A few drops of chloroform were added to the water bath to inhibit microbial growth. After dialysis, 5 mL of sample were filled into a tube and reacted with 1 mL of Alcian Blue (0.02% [w/v] in 0.06% acetic acid [v/v] [55], and adjusted to pH 2.5 with acetic acid. The mixture was vigorously mixed and left to stand for 5 min before mixing once more. The entire 6 mL were placed in a 10-mL syringe and filtered through a 0.2-μm-pore-size SFCA filter (Nalgene), and the last 1 mL of filtrate was measured at 610 nm against an ultra-high-purity (UHP) water blank using a spectrophotometer (Shimadzu UV 1700). The Alcian Blue side chains react with the acidic groups of polysaccharides yielding an insoluble non-ionic pigment, which is retained on the filter. The absorbance of the filtrate is inversely proportional to the concentration of DAPS in the sample. Two replicate samples were analyzed. It should be mentioned that only high molecular weight (>1kDa) DAPS can be determined with this method. DOM <1kDa will pass the dialysis membrane.

### Data analysis

Analysis of variance or Student’s *t*-test was conducted on the data that met the assumptions of normality and equality of variance. Data that did not meet these criteria were log (n + 1)-transformed before analyses, or nonparametric tests were carried out on the ranks. Nonparametric statistics (*Kolmogorov-Smirnov test*) was used to compare gels spatial–temporal differences. Tukey's honestly significant difference (Tukey HSD) test was used to compare the concentration of various substances at each station. Pearson product moment correlation analyze was used to examine the relationship among gels concentration and relevant environmental parameters. Principal component analysis (PCA) was employed to identify the key variables with the highest influence on parameters characteristics, directly on a correlation matrix. Statistical calculations were conducted using Origin9.0 (Origin Lab Corporation, USA).

## Results

### Physical parameters

**Fig 2:**
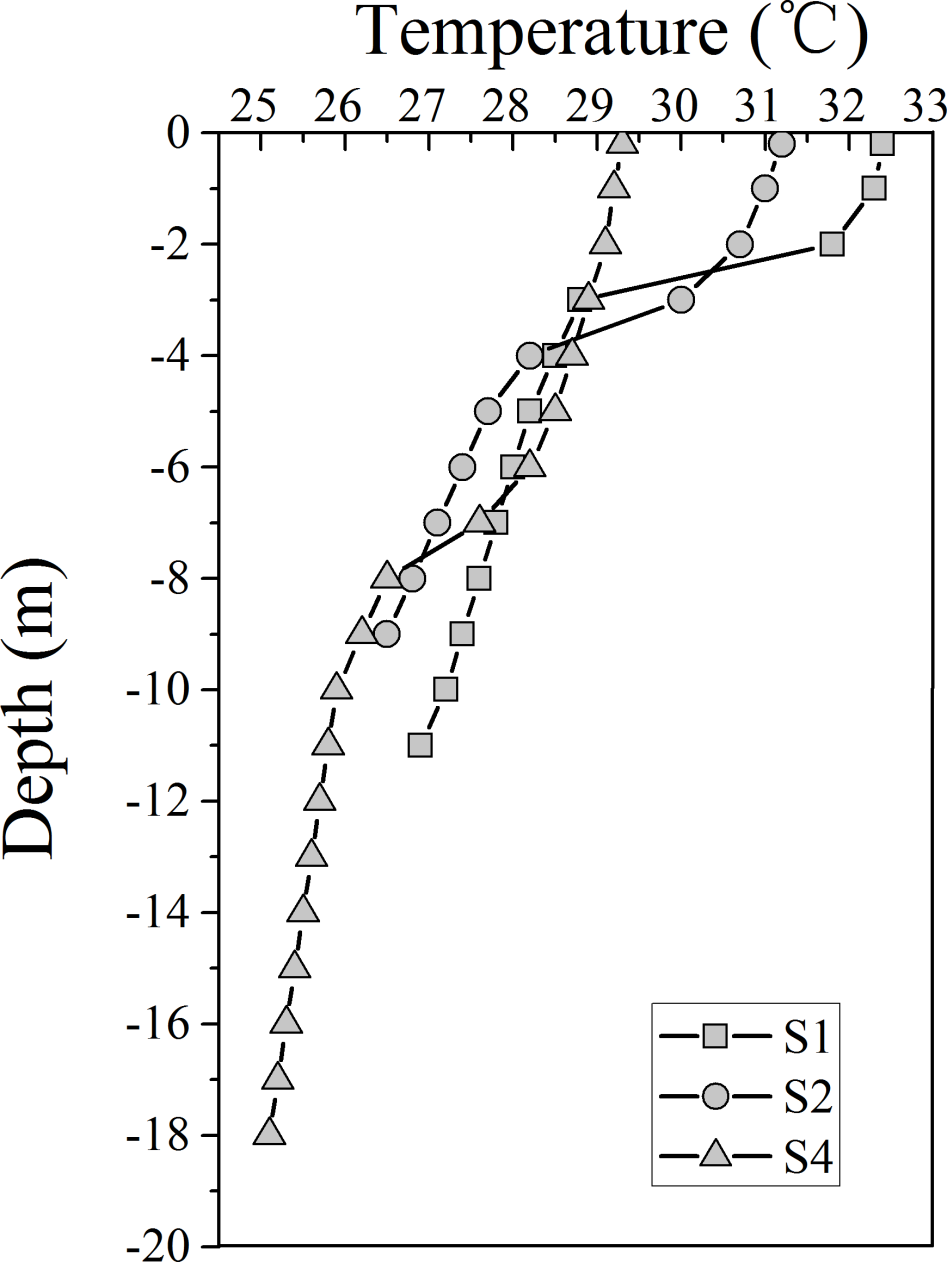
Vertical distribution of temperature at S1, S2 and S4.

Fig 1 showed the warm plume released from the LNpp and DNpp. S1 was the nearest station to the thermal outlet from Nuclear Power Plant during this cruise. Thus, the surface temperature at this site was the highest, and elevated about 3.0°C above lowest water temperature observed at S7 as shown in Fig 1 and Table 1. Moreover, the warm discharge from Npp resulted in the strongest stratification in temperature at S1 (Fig 2), where the difference of temperature between surface and bottom was 5.5°C. Temperature at S1 was relatively uniform in the upper 2 m, whereas the depth of thermocline at the other sites varied between 4 and 8m (Fig 2). This indicated that the thermocline in strength and depth varied spatially as a function of thermal discharge from Npp. The Southeast Asian southwesterly monsoon winds prevail with lower speed from May to September. Low wind speeds were recorded between 2.5 and 3.6 ms^-1^ during this cruise. The surface salinity ranged between 29.89 and 32.94 with the lowest salinity being observed at S3 (Table 1). During the cruise, the photosynthetically available radiation ranged from 740.5 to 1916.9 μmol m^-2^ s^-1^, and the maxima of UV radiation was 54.6 W m^-2^.

**Table 1:** Hydrographic conditions in Daya Bay.

Station	Geographic coordinates	Temperature(°C)		Salinity (‰)		Depth(m)
		Surface	Bottom	Surface	Bottom	
S1	114.5737° E, 22.6040° N	32.4	26.9	30.62	32.84	11.5
S2	114.5543° E, 22.5891° N	31.2	26.5	30.53	32.85	9.3
S3	114.5174° E, 22.5700° N	29.9	26.3	29.89	30.03	7.2
S4	114.6391° E, 22.5391° N	29.3	25.1	30.16	33.02	18.6
S5	114.5625° E, 22.5654° N	30.0	25.3	30.22	32.83	10.2
S6	114.6217° E, 22.6050° N	29.9	25.5	30.17	32.87	16.3
S7	114.6508° E, 22.6100° N	29.2	25.3	30.55	32.93	13.6

### Phytoplankton structure and nutrients

**Fig 3:**
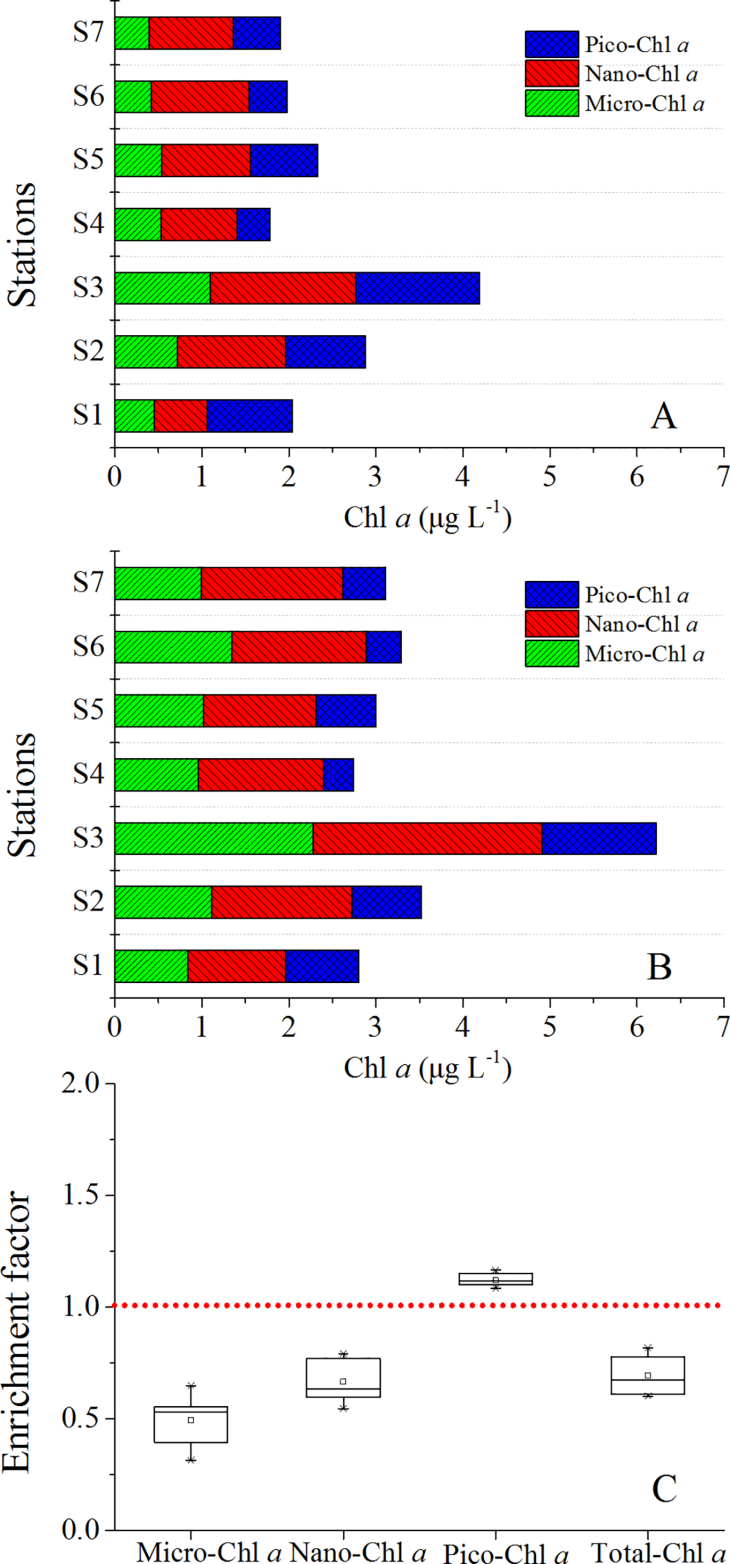
Size fractionation of Chl *a* in Daya Bay and enrichment factors. (A) Size fractionation of Chl *a* in the SML; (B) Size fractionation of Chl *a* in the SSL; (C) Enrichment factors of different size of Chl *a*.

**Fig 4:**
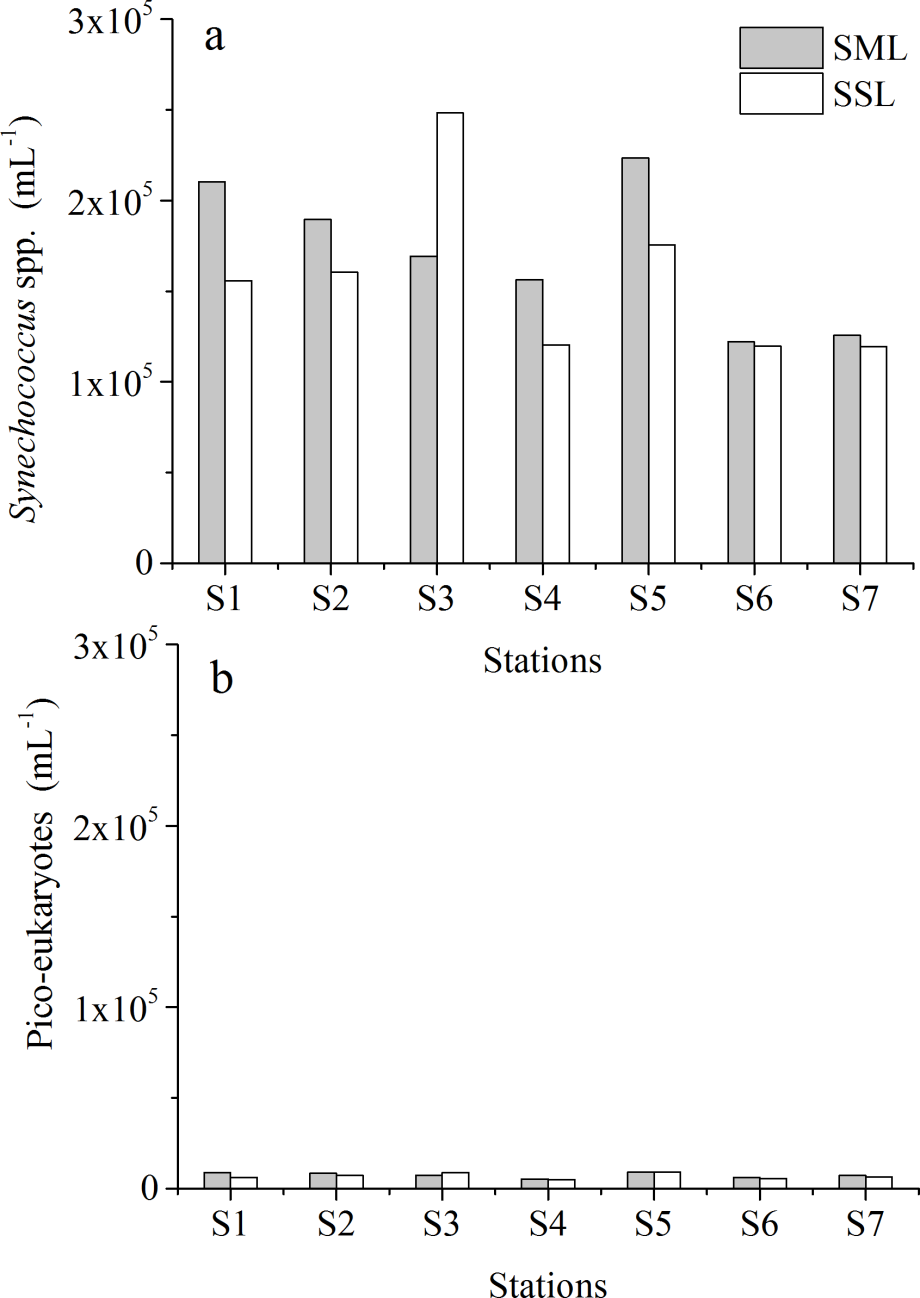
*Synechococcus* spp. and picoeukaryotes abundance in the SML and SSL in Daya Bay determined by flow cytometer. (a) *Synechococcus* spp. abundance; (b) picoeukaryotes abundance.

Total concentrations of Chl *a* varied between 1.78 and 4.19 μg L^-1^ in the SML (Fig 3A), and between 2.74 and 6.22 μg L^-1^ in the SSL (Fig 3B), respectively. In general, highest Chl *a* concentrations were observed at and close to the fish farming stations, (S2 and S3). Chl *a* concentration was dominated by nano-phytoplankton with an averaged contribution of 43%. Spatial variability of Chl *a* size fractions in the SML showed the highest contribution of pico-phytoplankton of 48% at S1 and the lowest contribution of 21% at S4, respectively (Fig 4A). Although there was depletion of total Chl *a* concentration, pico-phytoplankton were slightly accumulated in the SML (Fig 3C).

Abundances of picophytoplankton measured by flow cytometry are shown in Fig 4. *Synechococcus* spp. dominated bulk pico-phytoplankton abundance, whereas *Prochlorococcus* populations were not detected in this study. Pico-phytoplankton abundances varied between 1.19×10^5^ and 2.48×10^5^ 10mL^-1^ for *Synechococcus* spp. (Fig 4A) and between0.05×10^5^ and 0.09×10^5^ mL^-1^ for picoeukaryotes (Fig 4B). Highest abundance for *Synechococcus* spp. was observed at S3 for SSL samples and at S5 for the SML samples, respectively. The enrichment of pico-phytoplankton abundance differed spatially, with higher EF at the stations near the outlet of warm discharge from Npp (S1:EF_*Synechococcus spp*._ = 1.35, EF_*pico-euk*_ = 1.46) but depleted at S3 (EF _*Synechococcus spp*._ = 0.68, EF_*pico-euk*_ = 0.83).

**Fig 5:**
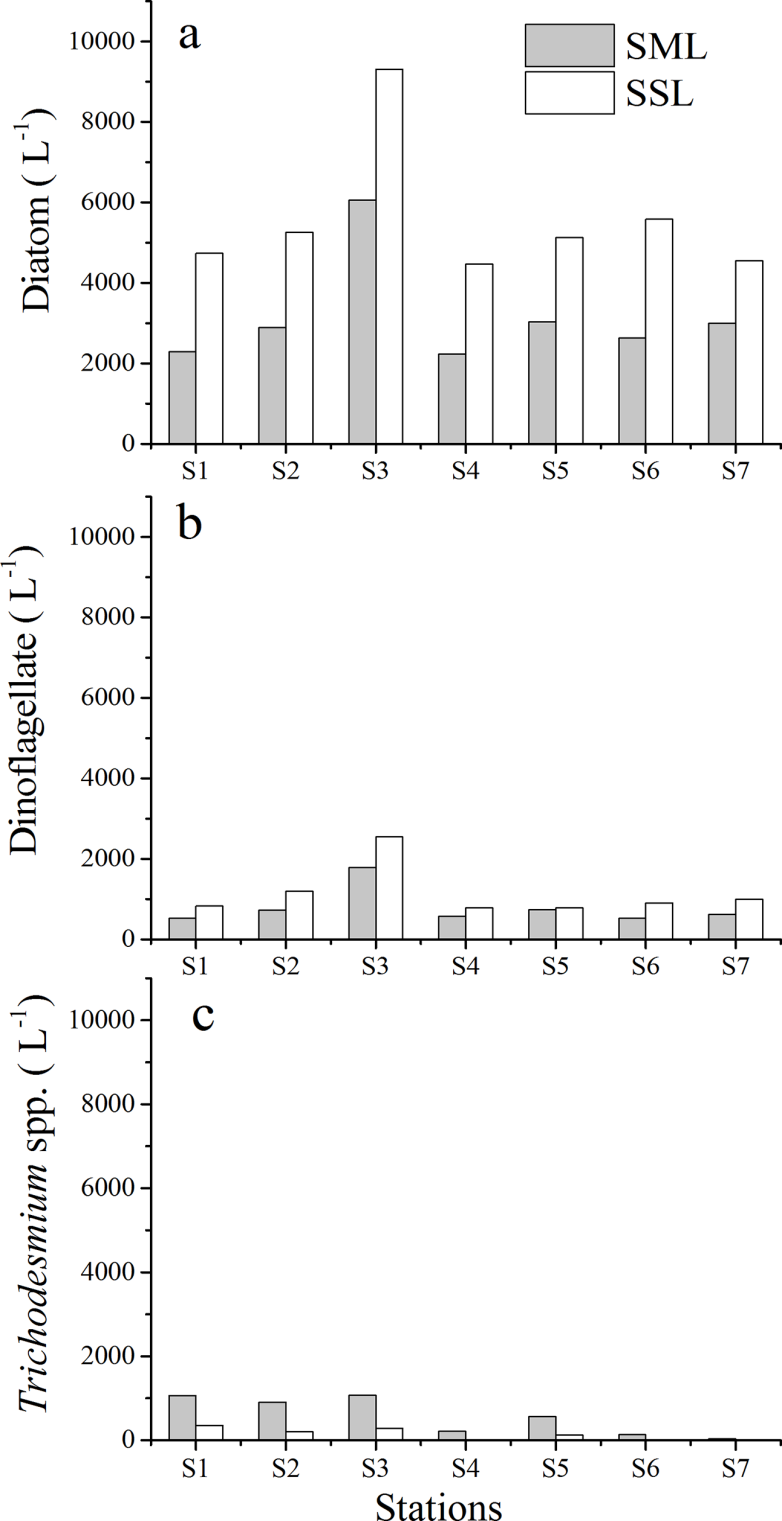
Phytoplankton abundance in the SML and SSL determined by light microscopy in Daya Bay. (a) Diatom abundance (cell L^-1^); (b) Dinoflagellate abundance (cell L^-1^); (c) *Trichodesmium* spp. abundance (filament L^-1^).

Phytoplankton abundance and community composition as determined by microscopy are shown in Fig 5A–5C. Phytoplankton cell densities ranged between 5.26×10^3^ to 12.1×10^3^ cells L^-1^ in the SSL with the mean of 6.86±2.38×10^3^ cells L^-1^. Diatoms, the dominant phytoplankton group, contributed 81.9% to total cell abundances in the SSL. The common dominant taxa included *Rhizosolenia delicatula*, *Rhizosolenia fragilissima*, and *Pseudo-nitzschia pungens*. Dinoflagellates, dominated by *Scrippsiella trochoidea*, represented 16.0% of total abundances; *Trichodesmium*, a widespread marine cyanobacterium with high nitrogen fixation properties was observed at S1, S2, S3 and S5. Clear differences were observed for the algal community between SML and underlying water determined in this study. Diatoms and dinoflagellates were clearly depleted in all SML samples (mean of EF _Diatoms_ = 0.56±0.07 and mean of EF _Dinoflagellates_ = 0.69±0.09). In contrast to the SSL, the SML had higher abundance and proportions of *Trichodesmium* spp., representing on average 12% of total phytoneuston abundance. Density of *Trichodesmium* spp.in the SML spatially varied from 36 to 1070 filaments L^−1^, with higher density at S1, S2 and S3 (Fig 5C).

Concentration of DIN was generally below 10 μmol L^-1^ in the SML and SSW, except for station S7 where DIN was 10.36 μmol L^-1^ in the SML (Table 2). The spatial distribution revealed that DIN concentrations were higher in the mouth of DYB and in the region far from the Npp discharge (S6 and S7). DIP concentration was on average 0.18 and 0.14 μmol L^-1^ in the SML and SSL, respectively. Ratios of DIN and DIP varied spatially from 20.7 to 54.7. Relatively low DIN/DIP ratios of about 20 were observed at S1 and S2. Nutrients were enriched in the SML on all stations, with EF’s ranging from 1.22 to 1.58 for DIP and from 1.24 to 1.52 for DIN (Table 2).

**Table 2:** Nutrients concentration in Daya Bay.

Stations		DIP [μmol L^-1^]	DIN [μmol L^-1^]	DIN/DIP
S1	SML	0.22	4.84	21.8
	SSL	0.18	3.77	20.7
	EF	1.22	1.29	
S2	SML	0.22	5.73	25.5
	SSL	0.17	3.76	21.8
	EF	1.30	1.52	
S3	SML	0.15	5.19	34.9
	SSL	0.12	3.44	29.5
	EF	1.27	1.51	
S4	SML	0.15	7.05	45.6
	SSL	0.10	5.36	54.7
	EF	1.58	1.32	
S5	SML	0.16	4.41	27.5
	SSL	0.12	3.56	29.0
	EF	1.31	1.24	
S6	SML	0.14	7.31	53.3
	SSL	0.10	4.99	48.3
	EF	1.33	1.47	
S7	SML	0.22	10.36	46.7
	SSL	0.17	7.60	44.1
	EF	1.29	1.36	

### Organic matter accumulation in the SML

**Fig 6:**
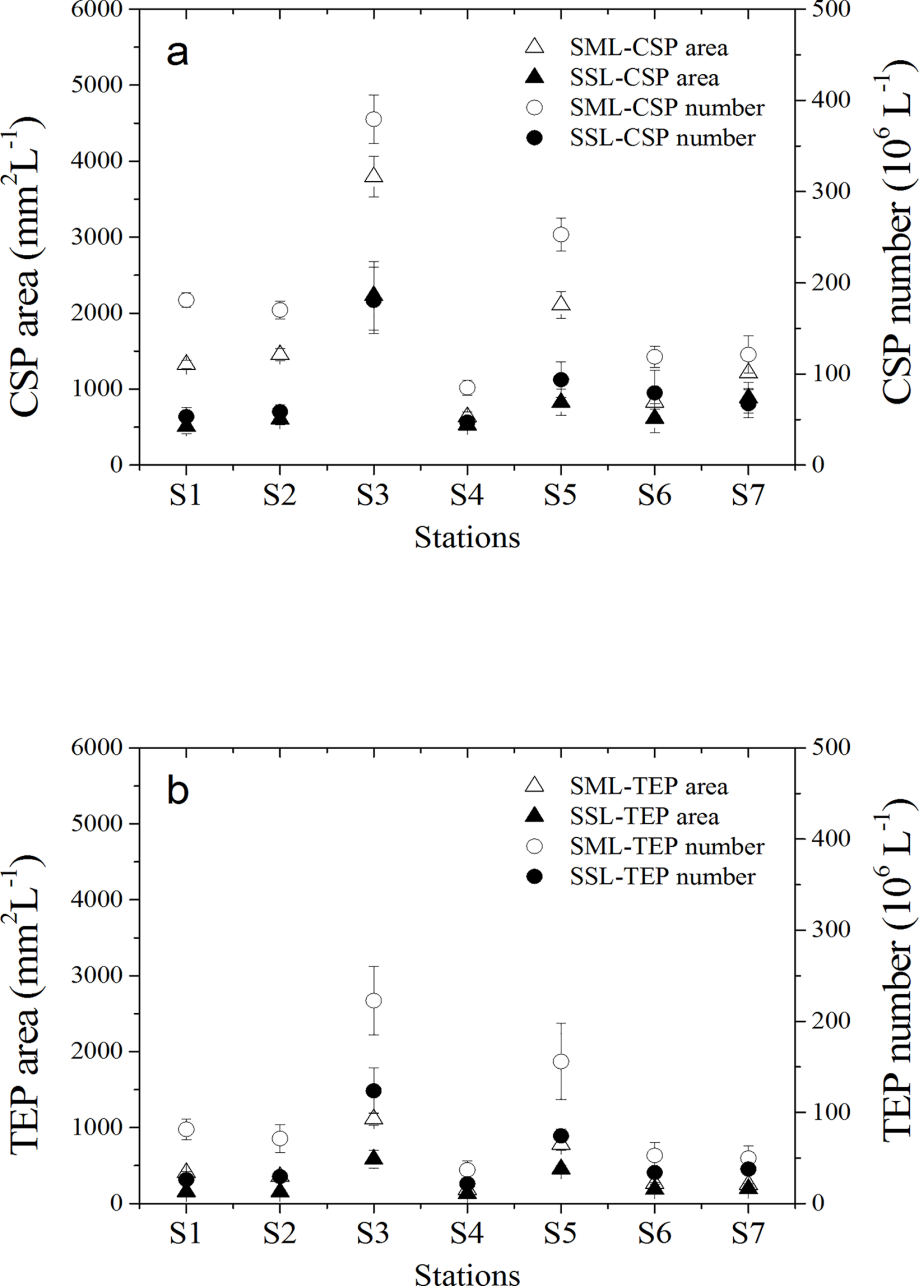
Abundance and total area distributions for TEP and CSP in the SML and SSL in Daya Bay. (a) CSP area and abundance; (b) TEP area and abundance.

**Fig 7:**
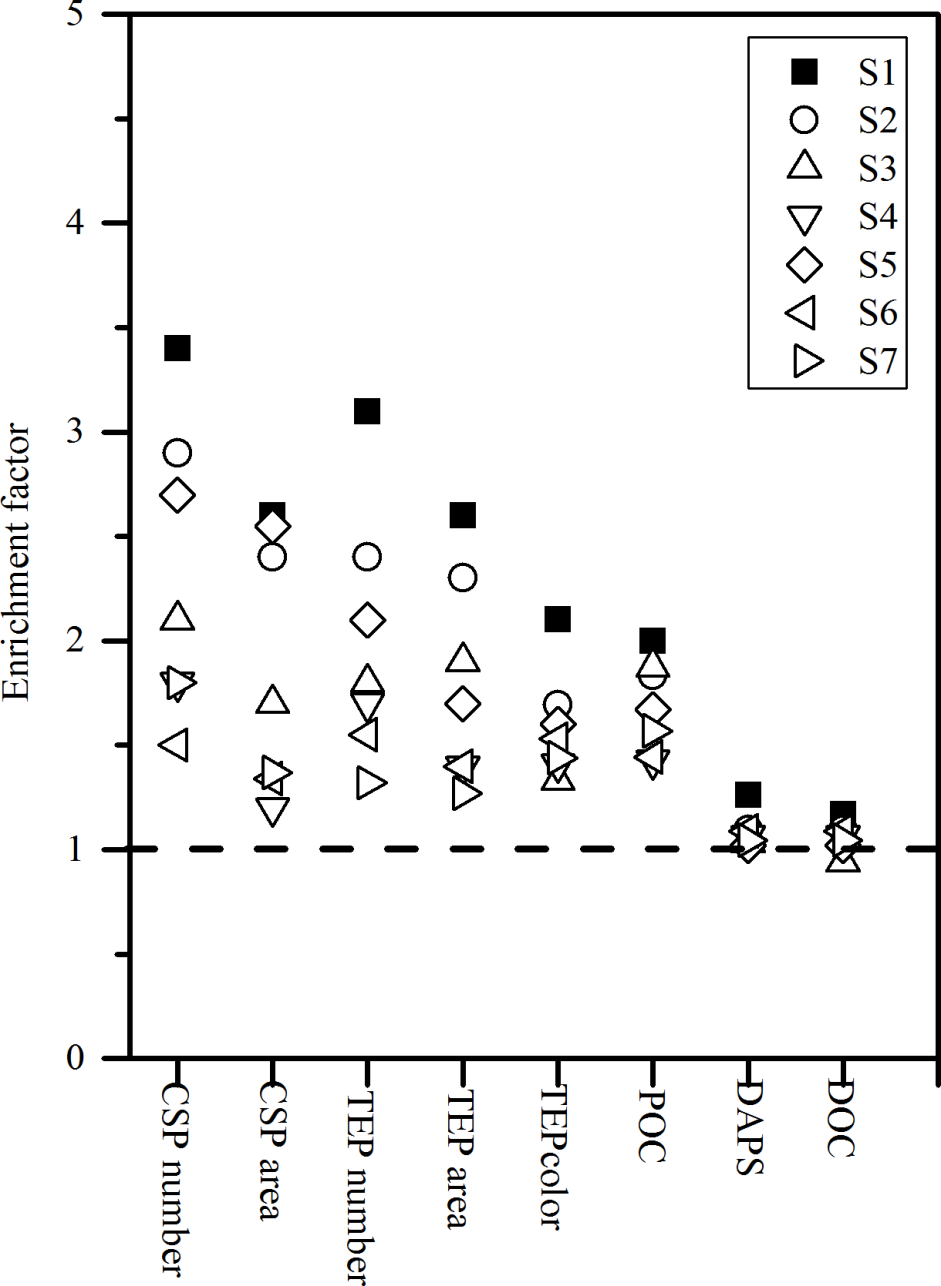
Enrichment factors for organic matter in the SML in Daya Bay.

Fig 6 shows the biogenic gels concentration in the SML and SSL. CSP abundance ranged from 84.9×10^6^ to 379×10^6^ L^-1^ in the SML and from 65.0 ×10^6^ to 181×10^6^ L^-1^ in the SSL. CSP numbers and total areas at S4, S6 and S7 were significant lower than those of the other stations (non-parameters t-test, p < 0.05). TEP abundance was clearly lower than CSP abundance on all stations, with a mean value of 95.9±60.7 ×10^6^ L^-1^ in the SML and 47.9±30.3×10^6^ L^-1^ in the SSL. Spatial variability of TEP abundance was similar to CSP abundance and yielded the highest value at S3 in the Dapeng bay. Abundance or total area of gels in the SML was significantly related to the respective concentration in the SSL (p<0.01). Thus, a similar pattern of spatial variability was observed for EF’s for abundance and total area of gel particles. EF’s varied from 1.5 to 3.4 for CSP numbers and from 1.32 to 3.2 for TEP numbers, respectively (Fig 7). A significant correlation was observed between the enrichment of microgels and temperature (p<0.05).

**Fig 8:**
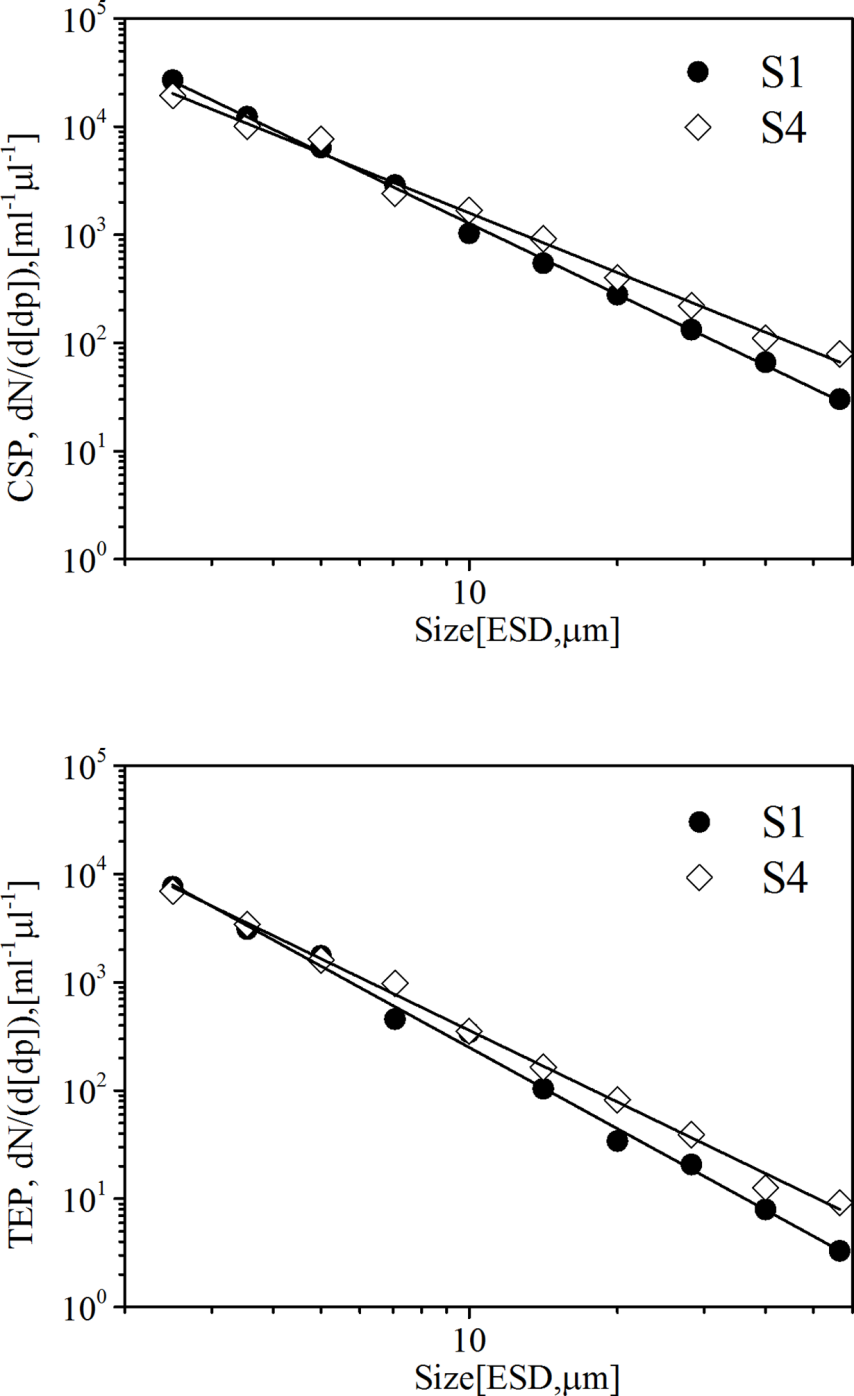
Size-frequency distribution of gel particles in the SML at S1 and S4.

The slope of the particles size spectrum could be described with the power law function given in Eq 3. The abundance in CSP and TEP in the SML followed the standard pattern of decrease in abundance with increasing particle size (r^2^_TEP_ = 0.99±0.01; r^2^_CSP_ = 0.96±0.02). The parameter δ describes the slope of the particles size spectra 2–60. For TEP, the slope varied from -2.49 to -2.19 (mean value: -2.30, SD: 0.11) for particles in the SML. The slope of CSP ranged from -2.18 to -1.83 (mean value:-1.95, SD: 0.12) in the SML. Lower values of slopes indicate relatively higher abundance of smaller particles. The slope of gels at S1 in the SML was significantly different from the other stations (p<0.05). The size distribution of gels with steepest slope for both CSP (δ = -2.18) and TEP (δ = -2.49) in the SML at S1 indicated a relatively higher abundance of smaller gels as shown in Fig 8, whereas maximum PSD slope was consistent with relatively more and larger-sized gels at the S4 where temperature was not affected totally by the Npp thermal discharge.

DOC concentrations ranged between 124.9 and 183.6 μmol L^-1^ and from 133.3 to 178.8 μmol L^-1^ in the SML and SSL, respectively (Table 3). For DOC at all stations, no enrichment was observed except for S1, showing a slight enrichment. Highest POC concentration water occurred at S3 both in the SSL and SML. In contrast to DOC, POC concentration was strongly enriched in the SML, at all stations. The average of DAPS concentrations were 2786.2±328.1μg L^−1^and 3017.8±288.8μg L^−1^ in the SML and SSL, respectively. A positive relationship was observed between DAPS and TEP_color_ concentrations (r^2^ = 0.678, p = 0.027). Similar to DOC, a relative enrichment of DAPS was more pronounced in SML of S1 (EFs = 1.26) than at other stations (Fig 7).

**Table 3:** Concentration of various organic components.

Stations	TEP_color_(μg Xeq L^−1^)		DAPS (μg L^−1^)		DOC (μmol L^−1^)		POC (μmol L^−1^)	
	SML	SSL	SML	SSL	SML	SSL	SML	SSL
S1	469.6±22.6	223.4±25.7	3131.7±272.5	2482.2±253.7	160.6±5.3	137.2±5.8	44.2	22.1
S2	548.5±51.7	324.2±30.1	3226.4±332.7	2947.6±299.0	178.8±7.7	163.3±7.8	48.6	26.5
S3	611.6±53.9	459.7±43.8	3397.8±364.1	3283.6±309.6	172.6±8.2	183.6±9.6	79.9	42.6
S4	270.1±22.4	191.6±20.5	2701.4±252.8	2531.8±247.9	133.3±7.3	124.9±5.7	28.4	19.9
S5	620.0±53.4	387.5±34.2	3189.9±306.9	3127.3±311.0	165.2±6.9	162.0±7.6	35.4	21.2
S6	488.4±45.2	319.2±31.6	2824.6±293.5	2591.4±244.3	167.9±7.2	154.1±8.9	34.2	23.7
S7	434.4±33.9	301.7±28.4	2653.6±255.1	2539.3±273.9	148.3±6.7	141.9±9.3	35.6	22.7

### Principal components analysis (PCA) for gels and environmental parameters

**Fig 9:**
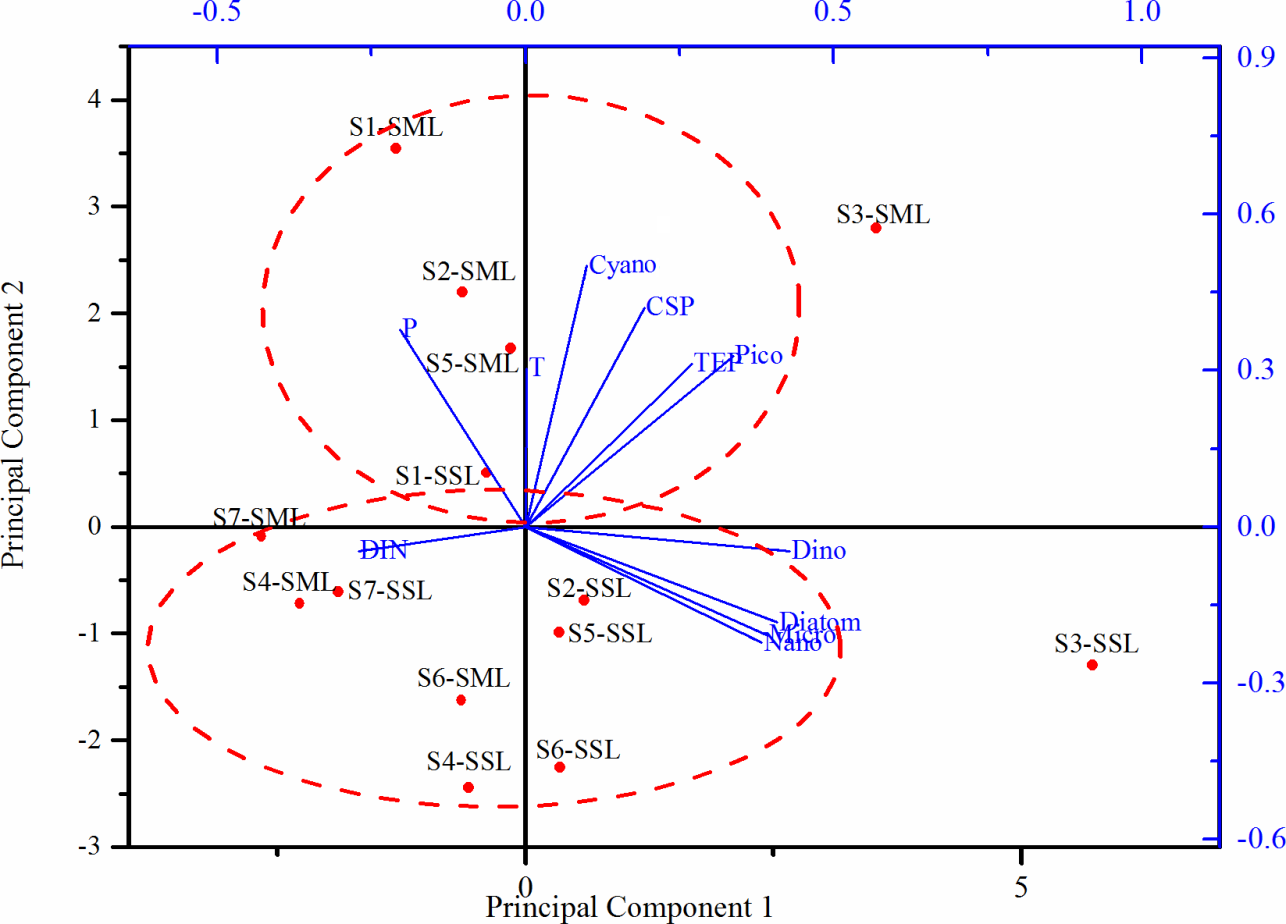
Ordination diagram displaying the first and second axes of principal components analysis (PCA). Loadings of the environmental and biological variables (DIN, dissolved inorganic nitrogen; P, dissolved inorganic phosphorus; Dino, Dinoflagellate; Cyano, cyanobacteria(mainly *Trichodesmium* spp.); Pico, Pico-size Chla; Nano, Pico-size Chla; Micro, Micro-size Chla) are displayed as blue line; the scheme for temporal variation of water parameters of samples depending on the scores of the first two principal components.

The relationships among the parameters, and spatial changes of biochemical parameters were further distinguished by PCA (Fig 9). The distribution of gel number was generally consistent with that of gel area, thus number was used in PCA. PCA showed that the first and the second principal components accounted for 44.75 and 31.85% of the total variability, respectively. Variability along the first axis was mainly explained by Chl-*a* concentration in the nano-size range, by diatom abundance as well as by CSP and TEP concentrations. It indicated that gel concentrations were related to the biomass. The second principal component (PC2), was associated with variables such as CSP, temperature, phosphate pico-Chla and cyanobacteria (mainly *Trichodesmium* spp.). It meant that the elevated temperature favored cyanobacteria and pico-phytoplankton growth. Among the investigated environmental variables, temperature, pico-size Chl *a* and cyanobacteria exhibited positive correlations with biogenic gels numbers in the SML. The samples could be divided into three subgroups: one group influenced by the warm discharge from Npp, with relatively higher temperature, phosphorus, high concentration of picoplankton Chl *a* and abundant gels in the SML; one group far from Npp was negatively related to PC1 and PC2 axes, with relative high DIN concentration. Samples from S3 distributed in the region of larger positive values of PC1, with relatively highest Chl *a* concentration in the micro- and nano-size classes.

## Discussion

The SML is a highly dynamic and heterogeneous layer with strong atmospheric influences. Wind speed is one of the primary factors that determines the enrichment and fate of particulate material in the SML [56–58]. Wind speed <5 ms^-1^ has little influence on the disruption of SML, due to the lack of near-surface mixing associated with a smooth surface without waves [57, 59]. In DYB, the SML is prone to formation of gelatinous films in summer, when wind speed is typically <5 m s^-1^ [60]. In this study, TEP and CSP were ubiquitous in the SML with EFs of 1.32–3.4. This is consistent with previous studies demonstrating that the SML is enriched in biogenic microgels [10, 16]. The wind speed during sampling was between 2.5–3.6 ms^-1^ supporting the accumulation of gels in the SML. Therefore, the effect of wind speed is not addressed in more detail in this study.

Data collected in this study showed that all biological and chemical components determined in the SML exhibited a close correlation with those in the underlying water, indicating that the SML reflects the ecosystem dynamics of the subsurface seawater system[16].

Many studies including biogeographic surveys, community-level experiments and single-species experiments indicate that average cell size of phytoplankton tend to become smaller in warmer waters [61–63]. The effects of temperature on size can be attributed both to direct temperature effects and to indirect effects mediated by nutrient supply [64, 65]. Stratification in DYB was observed from May to October with the mixing layer of 4-5m before onset of operation of the first Npp in 1980. It has been suggested that the warm discharge from Npp increases and prolongs the stratification at DYB [66]. During this study, temperature at the outlet of warm discharge decreased more rapidly with depth at 2m than waters farther away from the NPP. This suggested that the warm water discharge changed the vertical mixing regime in the proximity of the Npp. Furthermore, the lower level of DIN in the SML and SSL at S1 indicated that the increase in stratification could limit the nutrients vertical exchange across the pycnocline, or that nutrients were consumed more rapidly at higher temperature. This reduction in nutrient concentration may additionally shift community size to smaller species under warmer conditions [61, 67]. It has also been reported that low nutrient concentrations at higher temperatures affect the distribution diatom to a greater extent than small phytoplankton, such as picocyanobacteria [68]. Our results showed that the proportion of pico-Chl *a* to total Chl *a* varied spatially, with the highest contribution of pico-sized phytoplankton observed in the SML at S1 and S2, characterized by higher temperature, stronger stratification and low inorganic nitrogen concentrations relative to the samples collected far from the outlet of warm discharge. The observation of relative high enrichments of small picophytoplankton cells in the present study is similar with the findings of Wurl et al who reported increased abundance of total prokaryotic cells and picophytoplankton in slicks [4]. The reduction in plankton cell size could arguably result in a decrease of aggregate formation [69, 70], since coagulation rates, among others, are a function of particle number and size [35]. Thus, changes in plankton size could also affect the buoyancy and hence, potentially increasing their retention time and abundance of TEP in the surface.

EPS-induced DOC self-assembly and gel formation near the warm discharge can be stimulated via enhancement of hydrophobic interactions with temperature [71]. It has been suggested that temperature-induced conformational changes of amphiphilic polymers produce increased hydrophobic contact area and a higher probability of interchain bonding [71, 72]. In this study, the small size (0.4–2 μm) CSP and TEP contributed the majority of total microgels abundance in the SML at S1. It is in accordance with the observation that the equilibrium microgel size decreased with increased temperature [73]. In addition, strong pycnoclines due to warm discharge from Npp may slow-down settling speeds of aggregates [74], eventually supporting that biogenic gels particles experiences longer residence time in the upper water column at the Npp outlet. Also, the higher temperature may increase DOM, specifically carbohydrate exudation, leading to higher gel particles precursors [26]. Furthermore, higher temperature can increase the degradation rate of DOM and gel particles by heterotrophs [33]. Therefore, smaller gels observed at the vicinity of the Npp during this study may also indicate to enhanced heterotrophic degradation of larger gel particles.

*Synechococcus* spp. dominated pico-phytoplankton abundance during this study, corroborating previous findings that cynaobacteria like *Lyngbya*, *Oscillatoria* and *Synechococcus* spp., are important contributors to the total primary productivity in the SML in DYB [42]. Apart from *Synechococcus* spp., *Trichodesmium* spp. were observed only at the sites of S1, S2, S3 and S5, where the temperature was influenced by warm discharge. *Trichodesmium*, N-fixing cyanobacteria, have optimal growing conditions in warm, calm and stratified water [75, 76]. Thus, the environment conditions in DYB, characterized with persistent stratification and lower N:P ratios as well as high temperature during summer may favor cyanobacterial growth. Enrichment of *Synechococcus* spp. in the SML can also be attributed to an efficient adaption to UV-B stress by modifying the cellular photosystem [77, 78]. Changes in species composition may influence gel type and production[29, 79]. However, relatively little is known about exudation and gel particles formation in cyanobacteria communities compared to diatoms [80]. High light intensity was shown to increase the exudation of DON and DOC in cultures of *Nodularia spumigena*, with maximum exudates increase during the light period [81]. Additionally, observations of a pure culture of *Synechococcus bacillaris* by Cisternas-Novoa, Lee [79] showed that this cyanobacteria formed aggregates enriched in CSP. In our study, PCA indicated that gel numbers in the SML were positively correlated with temperature, cyanobacteria (mainly *Trichodesmium* spp.) and pico-Chl *a*. It is therefore speculated that the spatial distribution of gels in the SML of DYB can partly be explained by the presence of diazotrophic cyanobacteria and potentially increased nitrogen and carbon exudation stimulated by higher temperature. Although similar spatial pattern in abundance and total area were observed for CSP and TEP, these two type of gels showed different characteristics in DYB as concentrations in the SML were significantly higher for CSP than for TEP. Also, the slope of TEP was steeper than that of CSP, indicating more small TEP relative to CSP. Higher abundance of CSP compared to TEP has been observed previously at other marine sites [16, 82] and have been explained by CSP being less involved in aggregate formation and sinking out of the SML [16, 79].

It has been shown that high PAR and UVR could cleave DOC polymers, inhibit their spontaneous assembly, and/or disperse assembled microgels in SML [83], potentially affecting polymer dynamics in DYB. However, Mycosporine-like amino acids (MAAs) released from phytoplankton can be enriched in SML and exhibits an exponential rise in absorption in the UV as a protection against UV in the SML. In addition, particulate MAAs also can absorb UV radiation although in a narrow spectral band [84]. CSP may contain aromatic amino acids which can absorb UV light to different degrees [85]. No assessment was made, however, of whether CSP or amino acids accumulation in the SML could be a mechanism for UV attenuation due to the aromatic amino-acids present in the CSP.

## Conclusion

Our study showed that human influences, specifically seawater warming and eutrophication, affected the biogenic composition of the SML in DYB. Maximum concentration of gel particles and organic matter were observed in the Dapeng with high productivity, where water quality was impacted by the aquaculture and land nutrients load. Near the outlet of Npp, seawater experienced increased temperatures, more intense of thermal stratification. These environmental drivers combined with high UV radiation had substantial effects on phytoplankton species composition and biomass, especially favoring cyanobacteria over other phytoneuston and increasing contribution of pico phytoplankton to the total biomass in the SML. Inversely, diatom and dinoflagellate were depleted in the SML. Accumulation of gel particles differed spatially. Higher enrichment of gel particles and dissolved organic matter in the SML near the warm discharge outlet, and positive relationship between CSP and temperature and cyanobacteria suggested that gel particles in the SML were related to the increased temperature and coupled with the variation of phytoplankton activities, thus potentially influencing the biogeochemical cycling of nitrogen between the ocean and the atmosphere.

Reduction of gel size could increase the buoyancy and hence retention time of gels in surface waters[86], potentially increasing their abundance in the SML. A strong enrichment of TEP and CSP in submicron sea spray aerosol under field conditions has been observed [87]. Therefore, in the context of global warming, the decrease in gel size together with the increase in gel abundance in SML may favor organic aerosol formations, potentially changing the organic composition in the submicron sea spray aerosol. In addition, it seems more likely that in a warming scenario the impact of the SML on gas exchange may be higher since organics were in general more enriched at higher temperature. To better understand the role of temperature on the accumulation of microgels in the SML and the consequent potential for matter exchange across sea-air interface, future studies may need to address the relationship of microgels with other factors including bacterial effect, virus, and physical forcing, such as UV radiation and wind speeds. These are also important steps necessary to describe the effect of microgels on the process across sea-air interface in the context of higher temperature eventually.

## References

[1] Cunliffe M, Murrell JC. The sea-surface microlayer is a gelatinous biofilm. Isme J. 2009;3(9):1001–3. 10.1038/ismej.2009.69 PubMed PMID: WOS:000269618300001. 19554040

[2] Cunliffe M, Engel A, Frka S, Gasparovic B, Guitart C, Murrell JC, et al Sea surface microlayers: A unified physicochemical and biological perspective of the air-ocean interface. Prog Oceanogr. 2013;109:104–16. 10.1016/j.pocean.2012.08.004 PubMed PMID: WOS:000315059200008.

[3] Wurl O, Holmes M. The gelatinous nature of the sea-surface microlayer. Mar Chem. 2008;110(1–2):89–97. 10.1016/j.marchem.2008.02.009 PubMed PMID: WOS:000256715300009.

[4] Wurl O, Stolle C, Van Thuoc C, The Thu P, Mari X. Biofilm-like properties of the sea surface and predicted effects on air–sea CO2 exchange. Prog Oceanogr. 2016;144:15–24. 10.1016/j.pocean.2016.03.002.

[5] Long RA, Azam F. Abundant protein-containing particles in the sea. Aquat Microb Ecol. 1996;10(3):213–21. 10.3354/Ame010213 PubMed PMID: WOS:A1996UV15800001.

[6] Passow U. Transparent exopolymer particles (TEP) in aquatic environments. Prog Oceanogr. 2002;55(3–4):287–333. doi: Pii S0079-6611(02)00138-6 10.1016/S0079-6611(02)00138-6 PubMed PMID: WOS:000180210600002.

[7] Engel A, Thoms S, Riebesell U, Rochelle-Newall E, Zondervan I. Polysaccharide aggregation as a potential sink of marine dissolved organic carbon. Nature. 2004;428(6986):929–32. 10.1038/nature02453 PubMed PMID: WOS:000221083000037. 15118723

[8] Chin WC, Orellana MV, Verdugo P. Spontaneous assembly of marine dissolved organic matter into polymer gels. Nature. 1998;391(6667):568–72. PubMed PMID: WOS:000071842300046.

[9] Verdugo P. Marine Microgels. Annu Rev Mar Sci. 2012;4:375–400. 10.1146/annurev-marine-120709-142759 PubMed PMID: WOS:000300634900016. 22457980

[10] Wurl O, Miller L, Vagle S. Production and fate of transparent exopolymer particles in the ocean. J Geophys Res-Oceans. 2011;116. doi: Artn C00h13 10.1029/2011jc007342 PubMed PMID: WOS:000298758500002.

[11] Leck C, Bigg EK. Source and evolution of the marine aerosol—A new perspective. Geophys Res Lett. 2005;32(19). doi: Artn L19803 10.1029/2005gl023651 PubMed PMID: WOS:000232552100001.

[12] Orellana MV, Matrai PA, Leck C, Rauschenberg CD, Lee AM, Coz E. Marine microgels as a source of cloud condensation nuclei in the high Arctic. P Natl Acad Sci USA. 2011;108(33):13612–7. 10.1073/pnas.1102457108 PubMed PMID: WOS:000293895100053. 21825118 PMC3158224

[13] Simon M, Grossart HP, Schweitzer B, Ploug H. Microbial ecology of organic aggregates in aquatic ecosystems. Aquat Microb Ecol. 2002;28(2):175–211. 10.3354/Ame028175 PubMed PMID: WOS:000176872200007.

[14] Cunliffe M, Upstill-Goddard RC, Murrell JC. Microbiology of aquatic surface microlayers. Fems Microbiol Rev. 2011;35(2):233–46. 10.1111/j.1574-6976.2010.00246.x PubMed PMID: WOS:000286837600001. 20726895

[15] Gao Q, Leck C, Rauschenberg C, Matrai PA. On the chemical dynamics of extracellular polysaccharides in the high Arctic surface microlayer. Ocean Sci. 2012;8(4):401–18. 10.5194/os-8-401-2012 PubMed PMID: WOS:000309616400001.

[16] Engel A, Galgani L. The organic sea-surface microlayer in the upwelling region off the coast of Peru and potential implications for air-sea exchange processes. Biogeosciences. 2016;13(4):989–1007. 10.5194/bg-13-989-2016 PubMed PMID: WOS:000372082200008.

[17] Berman-Frank I, Spungin D, Rahav E, Van Wambeke F, Turk-Kubo K, Moutin T. Dynamics of transparent exopolymer particles (TEP) during the VAHINE mesocosm experiment in the New Caledonian lagoon. Biogeosciences. 2016;13(12):3793–805. 10.5194/bg-13-3793-2016 PubMed PMID: WOS:000379427700018.

[18] Galgani L, Stolle C, Endres S, Schulz KG, Engel A. Effects of ocean acidification on the biogenic composition of the sea-surface microlayer: Results from a mesocosm study. J Geophys Res-Oceans. 2014;119(11):7911–24. 10.1002/2014JC010188 PubMed PMID: WOS:000346102900031.

[19] Lass K, Bange HW, Friedrichs G. Seasonal signatures in SFG vibrational spectra of the sea surface nanolayer at Boknis Eck Time Series Station (SW Baltic Sea). Biogeosciences. 2013;10(8):5325–34. 10.5194/bg-10-5325-2013 PubMed PMID: WOS:000323980300005.

[20] Bar-Zeev E, Berman T, Rahav E, Dishon G, Herut B, Kress N, et al Transparent exopolymer particle (TEP) dynamics in the eastern Mediterranean Sea. Mar Ecol Prog Ser. 2011;431:107–18. 10.3354/meps09110 PubMed PMID: WOS:000291953100009.

[21] Grossart HP, Simon M, Logan BE. Formation of macroscopic organic aggregates (lake snow) in a large lake: The significance of transparent exopolymer particles, phytoplankton, and zooplankton. Limnol Oceanogr. 1997;42(8):1651–9. PubMed PMID: WOS:000073407700001.

[22] Engel A, Borchard C, Loginova A, Meyer J, Hauss H, Kiko R. Effects of varied nitrate and phosphate supply on polysaccharidic and proteinaceous gel particle production during tropical phytoplankton bloom experiments. Biogeosciences. 2015;12(19):5647–65. 10.5194/bg-12-5647-2015

[23] Kuznetsova M, Lee C, Aller J, Frew N. Enrichment of amino acids in the sea surface microlayer at coastal and open ocean sites in the North Atlantic Ocean. Limnol Oceanogr. 2004;49(5):1605–19. PubMed PMID: WOS:000224979900013.

[24] Carlucci AF, Wolgast DM, Craven DB. Microbial-Populations in Surface-Films—Amino-Acid Dynamics in Nearshore and Offshore Waters Off Southern California. J Geophys Res-Oceans. 1992;97(C4):5271–80. 10.1029/91jc02614 PubMed PMID: WOS:A1992HQ25600007.

[25] Kuznetsova M, Lee C. Enhanced extracellular enzymatic peptide hydrolysis in the sea-surface microlayer. Mar Chem. 2001;73(3–4):319–32. 10.1016/S0304-4203(00)00116-X PubMed PMID: WOS:000166821400009.

[26] Santos AL, Oliveira V, Baptista I, Henriques I, Gomes NCM, Almeida A, et al Effects of UV-B Radiation on the Structural and Physiological Diversity of Bacterioneuston and Bacterioplankton. Appl Environ Microb. 2012;78(6):2066–9. 10.1128/Aem.06344-11 PubMed PMID: WOS:000300629800055. 22247171 PMC3298163

[27] Borchard C, Engel A. Organic matter exudation by *Emiliania huxleyi* under simulated future ocean conditions. Biogeosciences. 2012;9(8):3405–23. 10.5194/bg-9-3405-2012

[28] Biermann A, Engel A, Riebesell U. Changes in organic matter cycling in a plankton community exposed to warming under different light intensities. J Plankton Res. 2014;36(3):658–71. 10.1093/plankt/fbu005

[29] Claquin P, Probert I, Lefebvre S, Veron B. Effects of temperature on photosynthetic parameters and TEP production in eight species of marine microalgae. Aquat Microb Ecol. 2008;51(1):1–11. 10.3354/ame01187 PubMed PMID: WOS:000256190600001.

[30] Engel A, Handel N, Wohlers J, Lunau M, Grossart HP, Sommer U, et al Effects of sea surface warming on the production and composition of dissolved organic matter during phytoplankton blooms: results from a mesocosm study. J Plankton Res. 2011;33(3):357–72. 10.1093/plankt/fbq122 PubMed PMID: WOS:000287025500002.

[31] Fukao T, Kimoto K, Kotani Y. Effect of temperature on cell growth and production of transparent exopolymer particles by the diatom Coscinodiscus granii isolated from marine mucilage. J Appl Phycol. 2012;24(2):181–6. 10.1007/s10811-011-9666-3 PubMed PMID: WOS:000300888200004.

[32] Seebah S, Fairfield C, Ullrich MS, Passow U. Aggregation and Sedimentation of Thalassiosira weissflogii (diatom) in a Warmer and More Acidified Future Ocean. Plos One. 2014;9(11). doi: ARTN e112379 10.1371/journal.pone.0112379 PubMed PMID: WOS:000344402600139. 25375640 PMC4223051

[33] Piontek J, Handel N, Langer G, Wohlers J, Riebesell U, Engel A. Effects of rising temperature on the formation and microbial degradation of marine diatom aggregates. Aquat Microb Ecol. 2009;54(3):305–18. 10.3354/ame01273 PubMed PMID: WOS:000264819200008.

[34] Kamykowski D, Zentara SJ. Changes in world ocean nitrate availability through the 20th century. Deep-Sea Res Pt I. 2005;52(9):1719–44. 10.1016/j.dsr.2005.04.007 PubMed PMID: WOS:000231589700008.

[35] Passow U, Carlson CA. The biological pump in a high CO2 world. Mar Ecol Prog Ser. 2012;470:249–71. 10.3354/meps09985 PubMed PMID: WOS:000311968200016.

[36] Wang YS, Lou ZP, Sun CC, Sun S. Ecological environment changes in Daya Bay, China, from 1982 to 2004. Mar Pollut Bull. 2008;56(11):1871–9. 10.1016/j.marpolbul.2008.07.017 PubMed PMID: WOS:000261362200019. 18783802

[37] Yin Jian-Ping WY-S, Xu Ji-Rong, Sun Cui-Ci, Zhang Feng-Qin. Seasonal thermocline in the Daya Bay and its influence on the environmental factors of seawater. Marine Science Bulletin. 2006;25(4):1–8.

[38] Wang YS, Lou ZP, Sun CC, Wang HL, Mitchell BG, Wu ML, et al Identification of water quality and zooplankton characteristics in Daya Bay, China, from 2001 to 2004. Environ Earth Sci. 2012;66(2):655–71. 10.1007/s12665-011-1274-7 PubMed PMID: WOS:000303413100024.

[39] Sun CC, Wang YS, Wu ML, Dong JD, Wang YT, Sun FL, et al Seasonal Variation of Water Quality and Phytoplankton Response Patterns in Daya Bay, China. Int J Env Res Pub He. 2011;8(7):2951–66. 10.3390/ijerph8072951 PubMed PMID: WOS:000293067300023. 21845168 PMC3155339

[40] Wu ML, Wang YS, Wang YT, Sun FL, Sun CC, Cheng H, et al Seasonal and spatial variations of water quality and trophic status in Daya Bay, South China Sea. Mar Pollut Bull. 2016;112(1–2):341–8. 10.1016/j.marpolbul.2016.07.042 PubMed PMID: WOS:000386188900049. 27491363

[41] Ma YE, Ke ZX, Huang LM, Tan YH. Identification of human-induced perturbations in Daya Bay, China: Evidence from plankton size structure. Cont Shelf Res. 2014;72:10–20. 10.1016/j.csr.2013.10.012 PubMed PMID: WOS:000329880000002.

[42] Wang HH, Song SH, Qi YZ. A comparative study of phytoneuston and the phytoplankton community structure in Daya Bay, South China Sea. J Sea Res. 2014;85:474–82. 10.1016/j.seares.2013.08.002 PubMed PMID: WOS:000329884700048.

[43] Tang DL, Kester DR, Wang ZD, Lian JS, Kawamura H. AVHRR satellite remote sensing and shipboard measurements of the thermal plume from the Daya Bay, nuclear power station, China. Remote Sens Environ. 2003;84(4):506–15. doi: Pii S0034-4257(02)00149-9 10.1016/S0034-4257(02)00149-9 PubMed PMID: WOS:000181734400003.

[44] Harvey GW, Burzell LA. Simple Microlayer Method for Small Samples. Limnol Oceanogr. 1972;17(1):156–&. PubMed PMID: WOS:A1972M759000020.

[45] Zhang Z, Liu L, Liu C, Cai W. Studies on the sea surface microlayer: II. The layer of sudden change of physical and chemical properties. J Colloid Interf Sci. 2003;264(1):148–59. 10.1016/S0021-9797(03)00390-4. 12885531

[46] GESAMP. The Sea-Surface Microlayer and its Role in Global Change. Reports and Studies. 1995.

[47] Herbland A, Lebouteiller A, Raimbault P. Size Structure of Phytoplankton Biomass in the Equatorial Atlantic-Ocean. Deep-Sea Res. 1985;32(7):819–36. 10.1016/0198-0149(85)90118-9 PubMed PMID: WOS:A1985AWD7000009.

[48] H SJD, R PT. A practical handbook of seawater analysis 1972.

[49] Engel A. Determination of Marine Gel Particles, in: Practical Guidelines for the Analysis of Seawater. 2009.

[50] Mari X, Kiorboe T. Abundance, size distribution and bacterial colonization of transparent exopolymeric particles (TEP) during spring in the Kattegat. J Plankton Res. 1996;18(6):969–86. 10.1093/plankt/18.6.969 PubMed PMID: WOS:A1996UU79900008.

[51] Jiang Q, Logan BE. Fractal Dimensions of Aggregates Determined from Steady-State Size Distributions. Environ Sci Technol. 1991;25(12):2031–8. 10.1021/Es00024a007 PubMed PMID: WOS:A1991GT52900013.

[52] Li XY, Zhang JJ, Lee JHW. Modelling particle size distribution dynamics in marine waters. Water Research. 2004;38(5):1305–17. 10.1016/j.watres.2003.11.010 PubMed PMID: WOS:000220012900025. 14975664

[53] Passow U, Alldredge AL. A dye‐binding assay for the spectrophotometric measurement of transparent exopolymer particles (TEP). Limnol Oceanogr. 1995;40(7):1326–35.

[54] Thornton DCO, Fejes EM, DiMarco SF, Clancy KM. Measurement of acid polysaccharides in marine and freshwater samples using alcian blue. Limnol Oceanogr-Meth. 2007;5:73–87. PubMed PMID: WOS:000246475800001.

[55] Passow U, Alldredge AL. Aggregation of a Diatom Bloom in a Mesocosm—the Role of Transparent Exopolymer Particles (Tep). Deep-Sea Res Pt Ii. 1995;42(1):99–109. 10.1016/0967-0645(95)00006-C PubMed PMID: WOS:A1995RF73100006.

[56] Liu KW, Dickhut RM. Effects of wind speed and particulate matter source on surface microlayer characteristics and enrichment of organic matter in southern Chesapeake Bay. J Geophys Res-Atmos. 1998;103(D9):10571–7. 10.1029/97jd03736 PubMed PMID: WOS:000073670500002.

[57] Romano JC. Sea-surface slick occurrence in the open sea (Mediterranean, Red Sea, Indian Ocean) in relation to wind speed. Deep-Sea Res Pt I. 1996;43(4):411–23. 10.1016/0967-0637(96)00024-6 PubMed PMID: WOS:A1996VE61800002.

[58] Sun C-C, Sperling M, Engel A. Effect of wind speed on the size distribution of biogenic gel particles in the sea surface microlayer: Insights from a wind wave channel experiment. Biogeosciences Discuss. 2017 10.5194/bg-2017-419.

[59] Wurl O, Wurl E, Miller L, Johnson K, Vagle S. Formation and global distribution of sea-surface microlayers. Biogeosciences. 2011;8(1):121–35. 10.5194/bg-8-121-2011 PubMed PMID: WOS:000286722500009.

[60] Wei X, Ni P, Zhan H. Monitoring cooling water discharge using Lagrangian coherent structures: A case study in Daya Bay, China. Mar Pollut Bull. 2013;75(1–2):105–13. 10.1016/j.marpolbul.2013.07.056. 23972680

[61] Moran XAG, Lopez-Urrutia A, Calvo-Diaz A, Li WKW. Increasing importance of small phytoplankton in a warmer ocean. Global Change Biol. 2010;16(3):1137–44. 10.1111/j.1365-2486.2009.01960.x PubMed PMID: WOS:000274419500018.

[62] Behrenfeld MJ, O'Malley RT, Siegel DA, McClain CR, Sarmiento JL, Feldman GC, et al Climate-driven trends in contemporary ocean productivity. Nature. 2006;444(7120):752–5. 10.1038/nature05317 PubMed PMID: WOS:000242581100059. 17151666

[63] Richardson AJ, Schoeman DS. Climate impact on plankton ecosystems in the Northeast Atlantic. Science. 2004;305(5690):1609–12. 10.1126/science.1100958 PubMed PMID: WOS:000223891200040. 15361622

[64] Shatwell T, Kohler J, Nicklisch A. Warming promotes cold-adapted phytoplankton in temperate lakes and opens a loophole for Oscillatoriales in spring. Global Change Biol. 2008;14(9):2194–200. 10.1111/j.1365-2486.2008.01630.x PubMed PMID: WOS:000258257700018.

[65] Sommer U, Peter KH, Genitsaris S, Moustaka-Gouni M. Do marine phytoplankton follow Bergmann's rule sensu lato? Biological Reviews. 2016:n/a-n/a. 10.1111/brv.12266 27028628

[66] Yin jP, Wang Y.S., Xu J.R., Sun C.C., Zhang F.Q. Seasonal Thermocline in the Daya Bay and its Influence on the Environmental Factors of Seawater. Marine Science Bulletin. 2006;25(4):1–8.

[67] Bopp L, Aumont O, Cadule P, Alvain S, Gehlen M. Response of diatoms distribution to global warming and potential implications: A global model study. Geophys Res Lett. 2005;32(19). doi: Artn L19606 10.1029/2005gl023653 PubMed PMID: WOS:000232685900003.

[68] Flombaum P, Gallegos JL, Gordillo RA, Rincon J, Zabala LL, Jiao NAZ, et al Present and future global distributions of the marine Cyanobacteria Prochlorococcus and Synechococcus. P Natl Acad Sci USA. 2013;110(24):9824–9. 10.1073/pnas.1307701110 PubMed PMID: WOS:000320930100056. 23703908 PMC3683724

[69] Burd AB, Jackson GA. Particle Aggregation. Annual Review of Marine Science. 2009;1:65–90. 10.1146/annurev.marine.010908.163904 PubMed PMID: WOS:000267421700004. 21141030

[70] Jackson GA, Burd AB. Aggregation in the marine environment. Environ Sci Technol. 1998;32(19):2805–14. 10.1021/Es980251w PubMed PMID: WOS:000076222600022.

[71] Ding YX, Chin WC, Rodriguez A, Hung CC, Santschi PH, Verdugo P. Amphiphilic exopolymers from Sagittula stellata induce DOM self-assembly and formation of marine microgels. Mar Chem. 2008;112(1–2):11–9. 10.1016/j.marchem.2008.05.003 PubMed PMID: WOS:000261824000002.

[72] Haidacher D, Vailaya A, Horvath C. Temperature effects in hydrophobic interaction chromatography. P Natl Acad Sci USA. 1996;93(6):2290–5. 10.1073/pnas.93.6.2290 PubMed PMID: WOS:A1996UB12100010. PMC39788 8637865

[73] Chen CS, Anaya JM, Chen EYT, Farr E, Chin WC. Ocean Warming-Acidification Synergism Undermines Dissolved Organic Matter Assembly. Plos One. 2015;10(2). doi: ARTN e0118300 10.1371/journal.pone.0118300 PubMed PMID: WOS:000350168700061. 25714090 PMC4340923

[74] Prairie JC, Ziervogel K, Arnosti C, Camassa R, Falcon C, Khatri S, et al Delayed settling of marine snow at sharp density transitions driven by fluid entrainment and diffusion-limited retention. Mar Ecol Prog Ser. 2013;487:185–200. 10.3354/meps10387 PubMed PMID: WOS:000322577400016.

[75] Carpenter EJ, Price CC. Nitrogen fixation, distribution, and production of Oscillatoria (Trichodesmium) spp. in the western Sargasso and Caribbean Seas1. Limnol Oceanogr. 1977;22(1):60–72. 10.4319/lo.1977.22.1.0060

[76] Douglas GC, Ajit S, Joseph PM, Maren V, Christoph H, Anne MJ, et al An extensive bloom of the N2-fixing cyanobacterium Trichodesmium erythraeum in the central Arabian Sea. Mar Ecol Prog Ser. 1998;172:281–92.

[77] Campbell D, Eriksson MJ, Oquist G, Gustafsson P, Clarke AK. The cyanobacterium Synechococcus resists UV-B by exchanging photosystem II reaction-center D1 proteins. P Natl Acad Sci USA. 1998;95(1):364–9. 10.1073/pnas.95.1.364 PubMed PMID: WOS:000071429500069. PMC18225 9419381

[78] Mohlin M, Roleda MY, Pattanaik B, Tenne SJ, Wulff A. Interspecific Resource Competition-Combined Effects of Radiation and Nutrient Limitation on Two Diazotrophic Filamentous Cyanobacteria. Microbial Ecol. 2012;63(4):736–50. 10.1007/s00248-011-9964-y PubMed PMID: WOS:000306127300004. 22057471

[79] Cisternas-Novoa C, Lee C, Engel A. Transparent exopolymer particles (TEP) and Coomassie stainable particles (CSP): Differences between their origin and vertical distributions in the ocean. Mar Chem. 2015;175:56–71. 10.1016/j.marchem.2015.03.009.

[80] Engel A, Meyerhofer M, von Brockel K. Chemical and biological composition of suspended particles and aggregates in the Baltic Sea in summer (1999). Estuar Coast Shelf S. 2002;55(5):729–41. 10.1006/ecss.2001.0927 PubMed PMID: WOS:000179546900005.

[81] Wannicke N, Koch BP, Voss M. Release of fixed N2 and C as dissolved compounds by Trichodesmium erythreum and Nodularia spumigena under the influence of high light and high nutrient (P). Aquat Microb Ecol. 2009;57(2):175–89.

[82] Thornton DCO, Brooks SD, Chen J. Protein and Carbohydrate Exopolymer Particles in the Sea Surface Microlayer (SML). Frontiers in Marine Science. 2016;3(135). 10.3389/fmars.2016.00135

[83] Orellana MV, Verdugo P. Ultraviolet radiation blocks the organic carbon exchange between the dissolved phase and the gel phase in the ocean. Limnol Oceanogr. 2003;48(4):1618–23. 10.4319/lo.2003.48.4.1618 PubMed PMID: WOS:000184247900025.

[84] Tilstone GH, Airs RL, Martinez-Vicente V, Widdicombe C, Llewellyn C. High concentrations of mycosporine-like amino acids and colored dissolved organic matter in the sea surface microlayer off the Iberian Peninsula. Limnol Oceanogr. 2010;55(5):1835–50. 10.4319/lo.2010.55.5.1835 PubMed PMID: WOS:000283667100003.

[85] Beaven GH, Holiday ER. Ultraviolet Absorption Spectra of Proteins and Amino Acids. Adv Protein Chem. 1952;7:319–86. 10.1016/S0065-3233(08)60022-4 PubMed PMID: WOS:A1952UM03900006. 14933256

[86] Mari X, Passow U, Migon C, Burd AB, Legendre L. Transparent exopolymer particles: Effects on carbon cycling in the ocean. Prog Oceanogr. 2017;151:13–37. 10.1016/j.pocean.2016.11.002.

[87] Aller JY, Radway JC, Kilthau WP, Bothe DW, Wilson TW, Vaillancourt RD, et al Size-resolved characterization of the polysaccharidic and proteinaceous components of sea spray aerosol. Atmos Environ. 2017;154:331–47. 10.1016/j.atmosenv.2017.01.053.

